# Exceptional Response to Second-Line Gemcitabine/Nab-Paclitaxel Chemotherapy in Patients With Metastatic Pancreatic Adenocarcinoma

**DOI:** 10.7759/cureus.18756

**Published:** 2021-10-13

**Authors:** Nedal Bukhari, Khalda Abdalla, Fahad Ibnshamsa, Waleed Alselwi, Shakir Al-Shakir, Mohammed Alqahtani

**Affiliations:** 1 Department of Medical Oncology, King Fahad Specialist Hospital, Dammam, SAU; 2 Department of Internal Medicine, Imam Abdulrahman Bin Faisal University, Dammam, SAU; 3 Department of Radiation Oncology, King Fahad Specialist Hospital, Dammam, SAU; 4 Multiorgan Transplant Center, King Fahad Specialist Hospital, Dammam, SAU

**Keywords:** chemotherapy, nab-paclitaxel, gemcitabine, folfirinox, pancreatic cancer

## Abstract

Pancreatic cancer is an extremely lethal malignancy with the majority of patients presenting with advanced disease. Typically, fit patients with advanced unresectable disease are treated with chemotherapy, which comprises either first-line folfirinox (FNX) or gemcitabine/nab-paclitaxel (GNP) regimens based on level 1 evidence. To our knowledge, robust evidence for second-line GNP post FNX does not exist. We herein report four cases treated at our institute with second-line GNP. Amongst those were patients with durable responses lasting over a year, which is extremely rare in stage 4 pancreatic cancer.

## Introduction

Pancreatic adenocarcinoma (PAC) is the eighth cause of cancer mortality and ranked the 14th in incidence worldwide in 2016 [1,2]. The current standard of care for resectable PAC is surgery followed by adjuvant chemotherapy. However, 15-20% of patients only present with potentially curative disease and the vast majority present with advanced unresectable disease, which is translated to a five-year survival rate of around 5% [3-5].

Over the last decade, new lines of treatments for metastatic PAC have emerged. Folfirinox (5-fluorouracil, leucovorin, irinotecan and oxaliplatin; FNX) and gemcitabine/nab-paclitaxel (GNP) both extended survival when compared to gemcitabine in first-line setting [6,7]. Second-line chemotherapy post-FNX therapy remains controversial due to the lack of phase III clinical trials. The best evidence comes from a phase II trial by Portal et al. where the median number of GNP cycles given to patients was four, with a median overall survival of 8.8 months [8]. We report four cases of metastatic PAC with durable responses to second-line GNP post-FNX. Three of them experienced a durable response lasting for 12 months and beyond, which is extremely rare in metastatic pancreatic cancer patients on palliative chemotherapy [9]. Those patients were treated at a tertiary hospital in Dammam, Saudi Arabia.

## Case presentation

Case 1

A 59-year-old male underwent Whipple’s procedure for a mass involving the head of the pancreas. Pathology was remarkable for a T3N1 poorly differentiated pancreatic ductal adenocarcinoma. Preoperative cancer antigen 19-9 (CA19-9) level was read at 282 U/ml. The patient received adjuvant capecitabine and gemcitabine (cape/gem) protocol for six months and was placed on surveillance afterwards.

**Figure 1:**
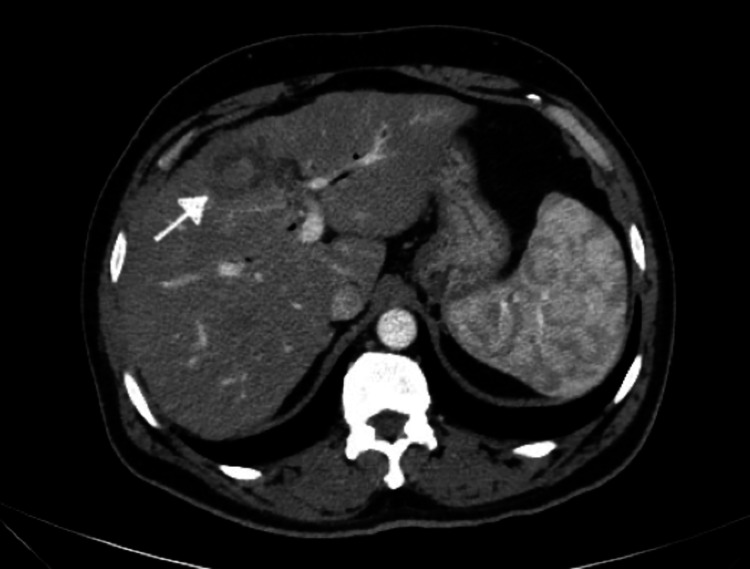
CT abdomen done prior to starting second-line gemcitabine/nab-paclitaxel (Case 1) The arrow indicates liver metastasis.

Seven months after completion of the adjuvant treatment, his computed tomography (CT) scan revealed recurrent disease involving the liver. He was started on palliative FNX every two weeks. Disease progression to his liver and retroperitoneal lymphadenopathy was confirmed on his CT scan after six cycles of FNX (Figure 1). He was then switched to second-line GNP immediately afterwards.

Before initiation of second-line GNP, his CA19-9 was at 1642.39, with a nadir of 6.8 U/ml. His tumor was tested for mismatch repair proteins and was found to be low in microsatellite instability (MSI-L).

**Figure 2:**
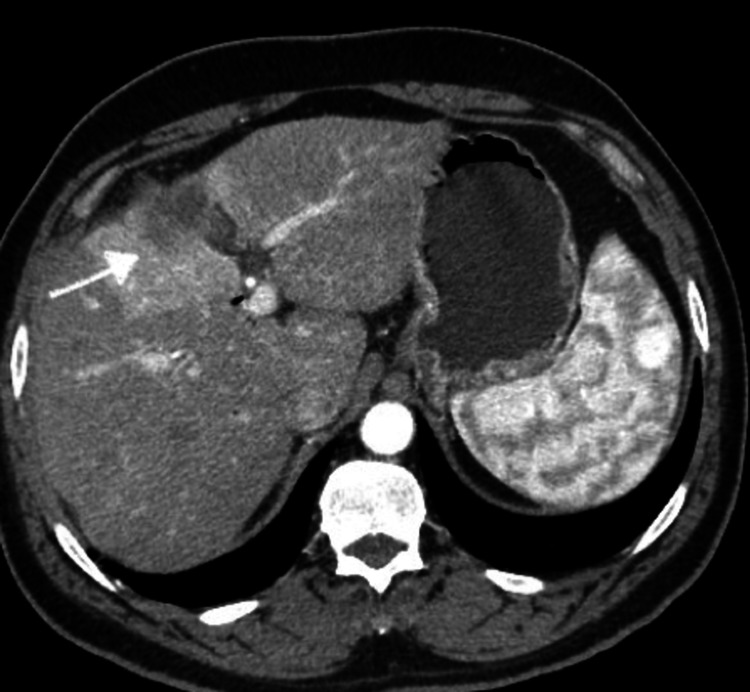
CT abdomen (Case 1) The figure indicates disease stability post 14 months of second-line gemcitabine/nab-paclitaxel.

He received a total of 14 cycles of GNP with multiple interruptions in his treatment course due to recurrent hepatic abscesses requiring long courses of intravenous (IV) antibiotics. His performance status dropped significantly after cycle 14 with an interval CT scan that showed interval disease progression with extensive liver metastasis (Figure 2). Second-line GNP was discontinued, and he was placed on complete palliative measures and died a few weeks afterwards (Table 1).

**Table 1:** Clinical Characteristics of Patients (Cases 1-4) **All patients received GNP in a 28-day cycle, where both nab-paclitaxel and gemcitabine were given on days 1, 8 and 15. GNP: gemcitabine/nab-paclitaxel; FNX: folfirinox; CA19-9: cancer antigen 19-9; MSI: microsatellite instability; ECOG: Eastern Cooperative Oncology Group; PS: performance status

Patients	PS pre GNP (ECOG)	Ca 19-9 pre GNP U/ml	Prior treatment and number of cycles	Number of GNP cycles*	Duration of response in months (m) while on GNP	Ca 19-9 nadir while on GNP U/ml	Grade III or higher side effects	Other molecular characteristics	Survival
Case 1	1	1642	FNX 6 cycles	14	18 m (patient had breaks from chemotherapy)	6.8	Not reported. Chemotherapy was interrupted	MSI-Low	19 m
Case 2	1	106	FNX 18 cycles	6	9 m	N/A	Fatigue and peripheral neuropathy	Unknown	10 m
Case 3	1	63.3	FNX 3 cycles only	9	12 m	4.08	Treatment interrupted	MS-Stable	12 m still alive on 3^rd^ line Folfiri
Case 4	1	662	Gemcitabine 4 cycles	14	14 m still receiving GNP with good clinical response	13.85	Nil	Unknown	16 m

Case 2

A 58-year-old male patient presented with metastatic pancreatic ductal adenocarcinoma, primary originating from the body and tail of the pancreas with metastatic paraaortic and aortocaval lymphadenopathy, peritoneal and bilateral pulmonary metastasis. At the time of presentation, his CA19-9 was at 359.96 U/ml.

He was started on palliative FNX with very good response to it lasting for over a year through which he received a total of 18 cycles. Treatment delays occurred several times, each lasting one to two weeks, mainly due to grade III fatigue and neutropenia. His CT scan done after 13 months indicated interval progression of his peritoneal metastasis and a new right adrenal gland metastasis. 

He was started on second-line GNP with a marked improvement in his disease reflected on interval CT scans done after three months. He received a total of six cycles of GNP over 10 months. 

Treatment delays occurred few times, mainly secondary to grade III fatigue and peripheral neuropathy. CA19-9 level prior to starting GNP was at 106.7 U/ml. His CA19-9 continued to climb up despite good clinical and radiological responses. The patient died of septic shock in ICU a few weeks after completion of cycle 6 (Table 1).

Case 3

**Figure 3:**
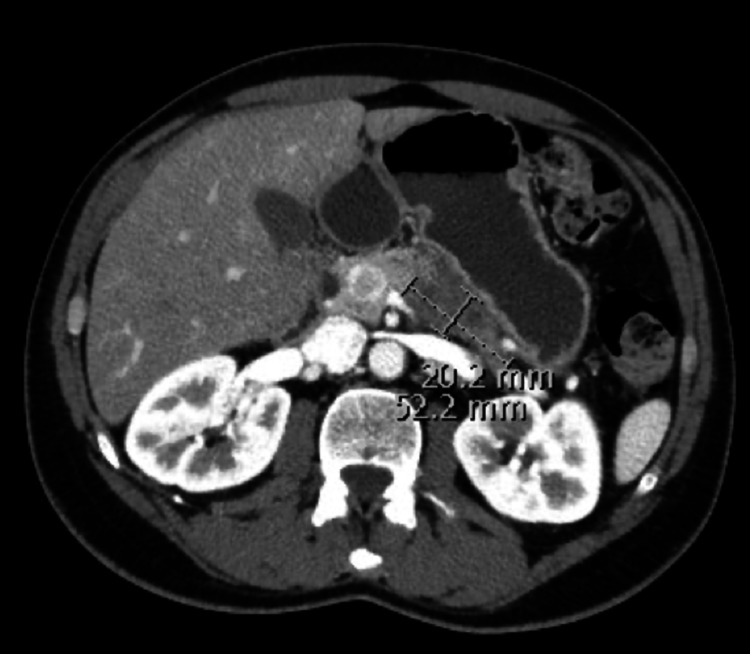
CT abdomen shows pancreatic mass prior to starting second-line GNP chemotherapy (Case 3) Arrows indicate the pancreatic mass

A 47-year-old male patient was referred from a community hospital with advanced pancreatic ductal adenocarcinoma, initially thought to be borderline resectable disease where three cycles of FNX were given. Disease progression involving the liver was noted on CT scan and curative surgery was declined (Figure 3). At the time of admission, his CA19-9 level was at 63U/ml. Due to interval disease progression, GNP was started. CA 19-9 nadir after four cycles was at 4.08 U/ml.

**Figure 4:**
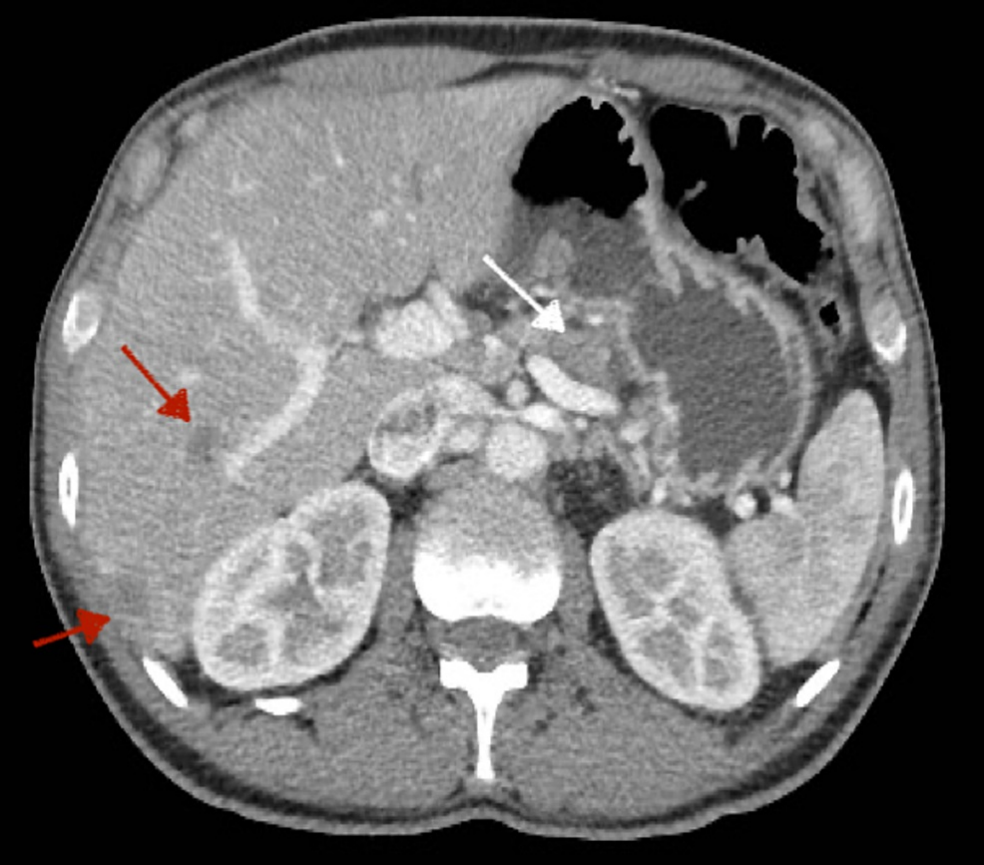
CT abdomen shows newly developed lesions in the liver (red arrows) after 12 months of starting second-line GNP (Case 3). The pancreatic mass showing interval regression (white arrow)

His CT done after 12 months of GNP showed interval disease progression of the primary lesion involving the body and tail of the pancreas with newly developed liver metastasis (Figure 4). He received a total of nine cycles of GNP throughout 12 months. His Medical Oncologist placed him on third-line Folfiri (5-FU, folinic acid and irinotecan) protocol due to interval clinical and radiological progression and he continues to be on it (Table 1).

Case 4

**Figure 5:**
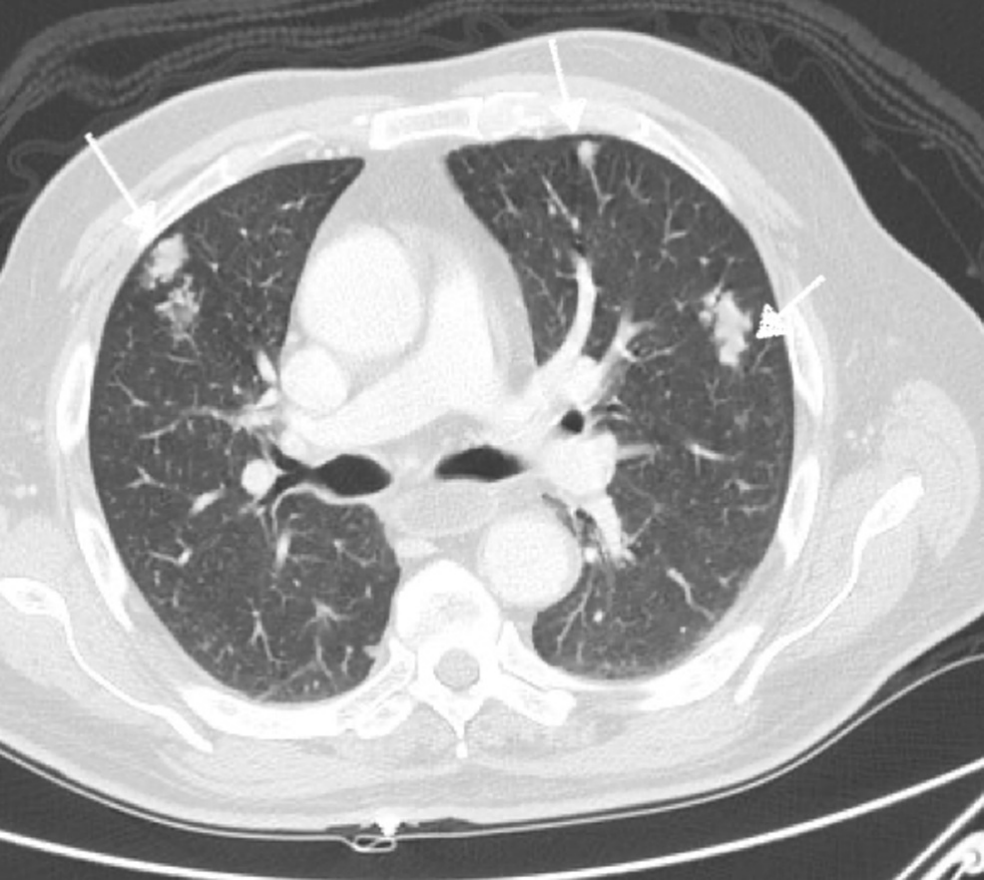
CT scan revealing bilateral pulmonary metastasis (Case 4). Arrows showing bilateral pulmonary metastatic lesions.

An 80-year-old male patient presented with pancreatic ductal adenocarcinoma with mediastinal lymphadenopathy and bilateral lung metastasis. His Eastern Cooperative Oncology Group (ECOG) performance status (PS) was borderline at 2. Therefore, he was started on single-agent gemcitabine with a marked improvement in his performance status after three cycles. However, his first reassessment CT scan done after cycle 3 revealed an interval progression of his pulmonary metastasis (Figure 5).

**Figure 6:**
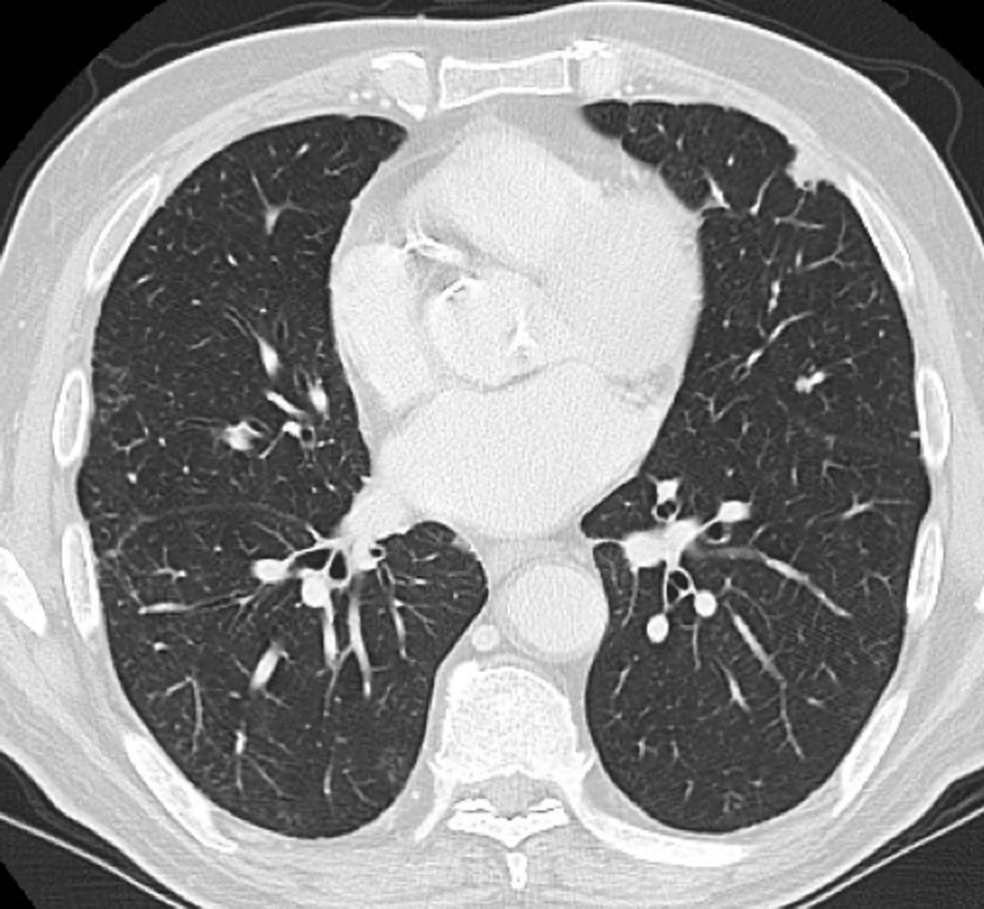
A reassessment CT scan done after 12 cycles of chemotherapy showing stable metastatic disease (Case 4). CT chest showing response to treatment.

His clinical improvement and performance status implied adding nab-paclitaxel to gemcitabine. A CT scan was done after 12 cycles of second-line GNP and showed stable metastatic disease (Figure 6). To date, he has completed a total of 14 cycles of GNP with plans to proceed further (Table 1).

## Discussion

Chemotherapy is the standard of care in metastatic PAC. FNX and GNP are both preferred, well-studied chemotherapeutic regimens in the first-line treatment of metastatic PAC.

A phase III trial of first-line FNX by Conroy et al. was the first to demonstrate a survival advantage closer to six months [6,10,11]. This regimen constitutes 5-fluorouracil, oxaliplatin, irinotecan and leucovorin and is usually offered to patients with an ECOG performance status of 0 to 1 [6,12]. Respectively, The MPACT trial clearly proved the overall survival (OS) advantage of GNP. This trial included patients with an ECOG PS of 2.7.

Second-line nano-liposomal irinotecan and infusional 5-FU showed a survival advantage in patients who progressed on first-line gemcitabine-based treatments [13]. To date, there is no standardized second-line treatment for metastatic pancreatic cancer (PC) patients progressing on first-line FNX due to the lack of conclusive level 1 evidence. However, there are small-scale phase II trials and observational studies that indicated objective response to second-line GNP, its safety and also a modest increase in OS [14].

We herein report four cases with an exceptional response to second-line GNP (Table 1) with Case 1 experiencing an 18-month survival after starting second-line GNP. Cases 1 and 2 experienced a prolonged response and OS to second-line GNP despite receiving prolonged FNX treatment courses in first-line setting, exceeding a year in Case 1. Case 4 had single agent gemcitabine initially due to borderline ECOG PS, for three months, commenced on GNP after a significant improvement in his PS, has been on this combination for 14 months with good response.

Over the last few years, extensive molecular and genomic research has been conducted to identify predictive and prognostic features in PC. Moffitt et al. identified two types of PC: classical, and basal-like type which carries the worst prognosis. The COMPASS trial showed that besides the typical histopathological characteristics, PC has different genomic and molecular subtypes with different responses to treatment classes [15,16]. The COMPASS trial clearly demonstrated that Moffit’s classical type expressed higher levels of GATA 6, responded better to first-line 5-FU-based chemotherapy, and also lived longer. Basal-like PC tumors were found to associate with low GATA 6 expression, resistance to adjuvant 5-FU based chemotherapy and demonstrated shorter OS. The predictive value of these classifications has not been investigated in adequately powered studies [15-17].

Approximately 10% of pancreatic malignancies harbour breast cancer (BRCA) gene mutations, detected by germlines testing and gene profiling of the tumor tissue. Fifty percent of those patients have germline BRCA and BRCA-like mutations in whom olaparib can be used as maintenance treatment post first-line platinum-based first line. Olaparib, a polyadenosine diphosphate-ribose polymerase (PARP) inhibitor was proven to prolong progression free survival (PFS) in a randomized phase III trial [18,19].

It is expected that germline mutations will confer the greatest benefit. However, a recent study indicated that PC harbouring germline or somatic mutations involving the homologous recombination repair (HRR) genes like BRCA1/2, PALB2, ATM, BAP1, BARD1, BLM, CHEK2, FANCA, BRIP1, FAM175A, FANCC, NBN, RAD50, RAD51, RAD51C and RTEL 1, will experience an improved PFS when treated with first-line platinum-based chemotherapy [18].

Microsatellite instability (MSI), present in approximately 1% of patients with PC, serves as a predictive biomarker for the anti-PD1 immunotherapy, pembrolizumab [18]. The phase II open label nonrandomized trial Keynote-158 enrolled patients with microsatellite instability-high (MSI-H) cancers including metastatic PAC patients who progressed on first-line standard chemotherapy. This trial concluded the efficacy and safety of pembrolizumab in MSI-H metastatic PAC. The subgroup analysis of PAC patients in this study showed an objective response rate (ORR) of 18.2%, a patient with complete response, three patients with partial response (PR) and a median OS of four months [20].

NTRK mutation is another good example of an actionable target, which is present in approximately 0.6% of patients with metastatic PC and may respond to anti-TRK agents like larotrectinib and entrectinib [21-23].

The main limitation of this series was the lack of information on molecular predictive and prognostic biomarkers, most of which are not routinely performed at our institute. Further studies are required to better understand the biology of pc and to identify and validate prognostic and predictive biomarkers that could help oncologists stratify and treat patients accordingly.

## Conclusions

This case series with patients demonstrating an exceptional response to second-line GNP supports the use of this line of treatment in metastatic PC patients progressing on first-line FNX. We also believe that current standards of care should incorporate further personalized medicine-based approaches to select the most appropriate treatments.

## References

[1] GBD 2017 Pancreatic Cancer Collaborators (2020). The global, regional, and national burden of pancreatic cancer and its attributable risk factors in 195 countries and territories, 1990-2017: a systematic analysis for the Global Burden of Disease Study 2017. Lancet Gastroenterol Hepatol.

[2] Kamisawa T, Wood LD, Itoi T, Takaori K (2016). Pancreatic cancer. Lancet.

[3] McGuigan A, Kelly P, Turkington RC, Jones C, Coleman HG, McCain RS (2018). Pancreatic cancer: a review of clinical diagnosis, epidemiology, treatment and outcomes. World J Gastroenterol.

[4] Dumont R, Puleo F, Collignon J (2017). A single center experience in resectable pancreatic ductal adenocarcinoma : the limitations of the surgery-first approach. Critical review of the literature and proposals for practice update. Acta Gastroenterol Belg.

[5] Labori KJ, Katz MH, Tzeng CW (2016). Impact of early disease progression and surgical complications on adjuvant chemotherapy completion rates and survival in patients undergoing the surgery first approach for resectable pancreatic ductal adenocarcinoma - a population-based cohort study. Acta Oncol.

[6] Conroy T, Desseigne F, Ychou M (2011). FOLFIRINOX versus gemcitabine for metastatic pancreatic cancer. N Engl J Med.

[7] Von Hoff DD, Ervin T, Arena FP (2013). Increased survival in pancreatic cancer with nab-paclitaxel plus gemcitabine. N Engl J Med.

[8] Portal A, Pernot S, Tougeron D (2015). Nab-paclitaxel plus gemcitabine for metastatic pancreatic adenocarcinoma after Folfirinox failure: an AGEO prospective multicentre cohort. Br J Cancer.

[9] Chae H, Jeong H, Cheon J (2020). Efficacy and safety of second-line nab-paclitaxel plus gemcitabine after progression on FOLFIRINOX for unresectable or metastatic pancreatic ductal adenocarcinoma: multicenter retrospective analysis. Ther Adv Med Oncol.

[10] Bukhari N, Winquist E (2017). Chronic oxaliplatin-based chemotherapy in a primary ampullary adenocarcinoma patient without significant peripheral neuropathy: case report and literature review. Case Rep Oncol.

[11] Bukhari N, Joudeh A (2019). Early stage anaplastic sarcomatoid carcinoma of the pancreas, a case report. Am J Case Rep.

[12] Azam F, Latif MF, Farooq A, Tirmazy SH, AlShahrani S, Bashir S, Bukhari N (2019). Performance status assessment by using ECOG (Eastern Cooperative Oncology Group) score for cancer patients by oncology healthcare professionals. Case Rep Oncol.

[13] Wang-Gillam A, Li CP, Bodoky G (2016). Nanoliposomal irinotecan with fluorouracil and folinic acid in metastatic pancreatic cancer after previous gemcitabine-based therapy (NAPOLI- 1): a global, randomised, open-label, phase 3 trial. Lancet.

[14] Mita N, Iwashita T, Uemura S (2019). Second-line gemcitabine plus nab-paclitaxel for patients with unresectable advanced pancreatic cancer after first-line FOLFIRINOX failure. J Clin Med.

[15] Moffitt RA, Marayati R, Flate EL (2015). Virtual microdissection identifies distinct tumor- and stroma-specific subtypes of pancreatic ductal adenocarcinoma. Nat Genet.

[16] Aung KL, Fischer SE, Denroche RE (2018). Genomics-driven precision medicine for advanced pancreatic cancer: early results from the COMPASS trial. Clin Cancer Res.

[17] Martinelli P, Carrillo-de Santa Pau E, Cox T (2017). GATA6 regulates EMT and tumour dissemination, and is a marker of response to adjuvant chemotherapy in pancreatic cancer. Gut.

[18] Park W, Chen J, Chou JF (2020). Genomic methods identify homologous recombination deficiency in pancreas adenocarcinoma and optimize treatment selection. Clin Cancer Res.

[19] Golan T, Hammel P, Reni M (2019). Maintenance olaparib for germline BRCA-mutated metastatic pancreatic cancer. N Engl J Med.

[20] Marabelle A, Le DT, Ascierto PA (2020). Efficacy of pembrolizumab in patients with noncolorectal high microsatellite instability/mismatch repair-deficient cancer: results from the phase II KEYNOTE-158 study. J Clin Oncol.

[21] Solomon JP, Linkov I, Rosado A (2020). NTRK fusion detection across multiple assays and 33,997 cases: diagnostic implications and pitfalls. Mod Pathol.

[22] O'Reilly EM, Hechtman JF (2019). Tumour response to TRK inhibition in a patient with pancreatic adenocarcinoma harbouring an NTRK gene fusion. Ann Oncol.

[23] Drilon A, Laetsch TW, Kummar S (2018). Efficacy of larotrectinib in TRK fusion-positive cancers in adults and children. N Engl J Med.

